# Metal ions in cerebrospinal fluid: Associations with anxiety, depression, and insomnia among cigarette smokers

**DOI:** 10.1111/cns.13955

**Published:** 2022-09-28

**Authors:** Yuying Li, Fenzan Wu, Qingshuang Mu, Kewei Xu, Shizhuo Yang, Ping Wang, Yuyu Wu, Junnan Wu, Wei Wang, Hui Li, Li Chen, Fan Wang, Yanlong Liu

**Affiliations:** ^1^ Ruian People's Hospital Wenzhou Medical College Affiliated Third Hospital Wenzhou China; ^2^ Laboratory of Translational Medicine Affiliated Cixi Hospital, Wenzhou Medical University Ningbo China; ^3^ School of Pharmacy Wenzhou Medical University Wenzhou China; ^4^ Xinjiang Key Laboratory of Neurological Disorder Research The Second Affiliated Hospital of Xinjiang Medical University Urumqi China; ^5^ School of Mental Health Wenzhou Medical University Wenzhou China; ^6^ Psychosomatic Medicine Research Division Inner Mongolia Medical University Huhhot China; ^7^ Department of Biomedical Engineering College of Engineering, Peking University Beijing China; ^8^ Beijing Hui‐Long‐Guan Hospital Peking University Beijing China; ^9^ The Affiliated Kangning Hospital Wenzhou Medical University Wenzhou China

**Keywords:** anxiety, cigarette smoking, depression, insomnia, metal ions

## Abstract

**Objective:**

The study aimed to investigate the relationship between cerebrospinal fluid (CSF) metal ions and anxiety, depression, and insomnia among cigarette smokers.

**Methods:**

We measured CSF levels of various metal ions from 178 Chinese male subjects. Apart from sociodemographic and clinical characteristics data, the Fagerstrom Test for Nicotine Dependence (FTND), Beck Depression Inventory (BDI), Self‐Rating Anxiety Scale (SAS), and Pittsburgh Sleep Quality Index (PSQI) were applied.

**Results:**

BDI and PSQI scores (all *p* < 0.001) were significantly higher in active smokers than nonsmokers. Active smokers have significantly higher CSF levels of magnesium, zinc, iron, lead, lithium, and aluminum (all *p* ≤ 0.002). Some metal ions, including zinc, iron, lead, and aluminum, were found to have a significant correlation with BDI scores, whereas metal ions, including zinc and lead, were found to have a significant correlation with PSQI scores in the general group. More interesting, mediation analysis showed that aluminum mediated the relationship between smoking and depression.

**Conclusions:**

Cigarette smoking was indeed associated with depression and insomnia. Active smokers had significantly higher CSF levels of magnesium, zinc, iron, lead, lithium, and aluminum. Furthermore, CSF aluminum played a mediating role in the relationship between smoking and depression, which further confirmed its neurotoxicity.

## Graphical abstract

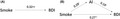

## 1 INTRODUCTION

China is the largest consumer of cigarettes, accounting for more than 40% of the total worldwide.^1^ Evidence from epidemiological studies and meta‐analyses showed that smoking might cause anxiety, depression,^2^, ^3^, ^4^ and insomnia.^5^, ^6^, ^7^ It has been reported that regular smoking was associated with an increased risk of anxiety and depression.^8^ Current smokers are more likely than nonsmokers to have sleep initiation and maintenance impairment, daytime sleepiness, and poor sleep quality.^7^, ^9^ These symptoms improve after quitting smoking.^2^ Nevertheless, the risk factors and the pathophysiology of anxiety, depression, and insomnia have not been fully understood.

Tobacco smoke contains about 4000 chemical substances, many toxic, especially nicotine^10^ and metals.^11^ Nicotine is the primary addictive substance. Studies showed that metal ions are associated with maintaining emotional homeostasis and circadian rhythm.^12^, ^13^ In addition, changes in these ions can cause emotional dysregulation and insomnia.^12^, ^13^, ^14^ Mengmei Ni^15^ and Falah S Al‐Fartusie et al.^16^ reported that the levels of aluminum and copper in the serum of patients with severe depression are significantly increased. Heavy metal ions (such as zinc, iron, lead, copper, and manganese) and other metal ions (such as lithium, aluminum, and magnesium) can be toxic in high concentrations, which may lead to neurophysiological changes.^14^

Many laboratories have developed evidence linking metal ions to anxiety, depression, and insomnia symptoms. Aluminum is the most common toxic mineral in the brain, lungs, liver, and thyroid.^17^ Studies showed that lesions in the hippocampus become more extensive and severe with brain aluminum content, inducing nerve cells apoptosis and necroptosis, which contribute to the deterioration of psychological function, including depression.^18^, ^19^ Besides, excess manganese preferentially accumulates in mitochondrial‐rich tissues, which is neurotoxic. Studies showed that manganese exposure might induce severe anxiety, depression,^20^ and sleep disorder.^21^ Zinc and iron are abundant trace elements in the human central nervous system (CNS). The mechanisms of zinc‐mediated neurotoxicity appear to include not only neuronal signaling but also the regulation of mitochondrial function and energy production.^22^ However, there is no consensus on the effect of zinc overload or zinc deficiency in anxiety, depression, and insomnia. Likewise, both iron deficiency and iron overload can affect the redox state, and the associations between dietary iron intake and risk of sleep disorder remain controversial.^23^, ^24^

Cigarette smoke enters the brain quickly by many pathways and may cause metal ions to accumulate. However, no study has been published yet that uses cerebrospinal fluid (CSF) metal ions to investigate the relationship between active smoking and emotional dysfunction and sleep disorder. Therefore, this study aimed to determine associations between cigarette smoking and anxiety, depression, and insomnia by measuring CSF metal ions.

## 2 MATERIALS AND METHODS

### 2.1 Participants

Because few women smoke in China (3.2%), we considered only Chinese male subjects.^25^ A total of 178 Chinese subjects scheduled for anterior cruciate ligament reconstruction surgery were recruited from September 2014 to January 2016, as described.^26^ Of these, 64 were active smokers, while 114 were nonsmokers. We recorded sociodemographic data, including age, years of education, and body mass index (BMI). Clinical data, including a history of substance abuse and dependence, were obtained according to self‐report and confirmed by the next of kin and family members. Exclusion criteria were as follows: (1) a family history of psychosis or neurological diseases; and (2) systemic or CNS diseases determined by the Mini‐International Neuropsychiatric Interview.

Participants who had never smoked and had no history of substance abuse or dependence were assigned to the nonsmokers' group. Active smokers were defined as those who had consumed half a pack of cigarettes (i.e., 10 cigarettes) or more per day for more than 1 year, according to the Diagnostic and Statistical Manual of Mental Disorders, 4th Edition. Smokers who consumed fewer than 10 cigarettes per day were excluded. No participants had a history of alcohol abuse or psychiatric disorders in our study. The Institutional Review Board of Inner Mongolian Medical University approved the study, performed following the Declaration of Helsinki, and written informed consent was obtained.

### 2.2 Assessments, biologicals sample collection, and laboratory tests

Each questionnaire was the Chinese version. The Fagerstrom Test for Nicotine Dependence (FTND) is the most common scale for assessing nicotine dependence (ND) and is translated from the FTND assessment.^27^ A score of 1–3 is categorized as low ND, 4–6 is medium ND, and 7–10 is high ND. The Pittsburgh Sleep Quality Index (PSQI) measures sleep quality over the last month. According to the instruction of the Chinese version of PSQI,^28^ participants are asked to respond on a four‐point Likert scale (from 0 = “no difficulty” to 3 = “severe difficulty”). Items are combined to form seven subscales: subjective sleep quality, latency, duration, habitual efficiency, disturbances, use of sleep medication, and daytime dysfunction. The sum of the response scores can range from 0 to 21. The higher the score, the worse the sleep quality. The Beck Depression Inventory (BDI) and Self‐Rating Anxiety Scale (SAS) were used for emotion assessment.^29^ The BDI and SAS scores for all subjects were less than 17 and 50, respectively. The subjects completed these assessments by self‐report 1 day before CSF extraction. Information regarding smoking‐related habits was obtained from active smokers: age at onset, years of cigarette smoking, the average daily amount of cigarette smoking, and maximum daily amount of cigarette smoking.

Lumbar puncture is part of China's standard clinical procedure for patients undergoing anterior cruciate ligament reconstructive surgery, making the CSF sample conveniently accessible and decreasing the likelihood of the effect of disease entity on the CSF sample. Preoperative smoking cessation is not required for this kind of operation. In this study, a licensed anesthetist conducted a lumbar puncture in the morning before surgery using 3 ml of 0.5% ropivacaine as local anesthesia for all subjects, and a 5‐ml CSF sample was obtained via intrathecal collection. The samples were immediately frozen at −80°C. It takes less than 1 hour to complete the entire anterior cruciate ligament reconstruction. The time from subject hospitalization to surgery was a maximum of 2 days.

Using atomic absorption spectrophotometry, the analyses were performed to measure CSF levels of magnesium, zinc, iron, lead, lithium, copper, manganese, and aluminum. Laboratory technicians were blinded to clinical data.

### 2.3 Statistical analysis

The normality of all variables was assessed using the Shapiro–Wilk test. Only zinc, iron, and aluminum were normally distributed (*p* > 0.05). Consequently, the Mann–Whitney rank‐sum test was used to compare differences in general demographic data, clinical data, and raw biomarkers between groups. Spearman correlation analysis was performed to test the relationship between CSF metal ion levels and scores. Then, adjusting for age, years of education, and BMI, a mediation effect analysis was performed to clarify whether metal ions mediated the relationship between smoking and scores. All statistical analyses were performed using IBM SPSS Statistics for Windows, Version 22.0 (IBM Corp., Armonk) and Process macro program (V3.4 by Andrew F.). Figures were created using the R Programming Language 4.2.0. All tests were two‐sided, and the significance threshold was set at *p* < 0.05.

## 3 RESULTS

### 3.1 Sociodemographic and clinical characteristics

The results are displayed in Table 1. BMI (25.70 ± 3.40 vs. 24.37 ± 4.17, *p* = 0.01), BDI, and PSQI scores (3.65 ± 4.89 vs. 1.29 ± 2.15, *p* < 0.001 and 4.53 ± 2.69 vs. 2.79 ± 2.56, *p <* 0.001) were significantly higher in active smokers than that in nonsmokers. Significantly higher CSF levels of magnesium, zinc, iron, lead, lithium, and aluminum (all *p* ≤ 0.002) were found in active smokers than in nonsmokers, consistent with our previous study.^28^

**TABLE 1:** Characteristics differences between nonsmokers and active smokers

Variables	Nonsmokers	Active smokers	*p*
	(*n* = 104) (Mean ± SD) (Median, IQR)	(*n* = 57) (Mean ± SD) (Median, IQR)	
Age (years)	29.61 ± 9.26 (28.00, 15)	32.49 ± 9.35 (31.00, 11)	0.05
Education (years)	13.04 ± 2.68 (15.00, 4)	12.05 ± 3.47 (15.00, 7)	0.12
***BMI***	24.37 ± 4.17 (23.58, 5.01)	25.70 ± 3.40 (25.51, 4.78)	***0*.*01*** ^*^
SAS	33.58 ± 4.40 (35.00, 4)	34.35 ± 4.88 (35.00, 5.50)	0.28
***BDI***	1.29 ± 2.15 (0.00, 2.00)	3.65 ± 4.89 (2.00, 3.00)	***<0*.*001*** ^*^
***PSQI***	2.79 ± 2.56 (2.00, 3.00)	4.53 ± 2.69 (4.00, 3.00)	***<0*.*001*** ^*^
***Mg(mmol/L)***	0.75 ± 0.48 (0.65, 0.40)	0.91 ± 0.20 (0.88, 0.23)	***<0*.*001*** ^*^
***Zn(μmol/L)***	11.34 ± 1.40 (11.24, 1.95)	13.05 ± 1.49 (12.84, 2.25)	***<0*.*001*** ^*^
***Fe(μmol/L)***	11.26 ± 1.63 (11.24, 2.25)	14.54 ± 1.23 (14.29, 1.64)	***<0*.*001*** ^*^
***Pb(μg/L)***	119.52 ± 17.36 (123.53, 18.91)	139.30 ± 13.02 (137.29, 18.21)	***<0*.*001*** ^*^
***Li(mmol/L)***	0.57 ± 0.17 (0.55, 0.27)	0.67 ± 0.12 (0.63, 0.16)	***<0*.*001*** ^*^
Cu(mg/L)	0.67 ± 0.12 (0.67, 0.12)	0.67 ± 0.08 (0.67, 0.12)	1.00
Mn(μmol/L)	0.03 ± 0.01 (0.03, 0.01)	0.03 ± 0.01 (0.03, 0.01)	0.11
***Al(μmol/L)***	0.88 ± 0.09 (0.88, 0.13)	0.93 ± 0.09 (0.93, 0.10)	***0*.*002*** ^*^
FTND	(−)	3.44 ± 2.27 (4.00, 3.50)	(−)
Age of smoking onset (years)	(−)	20.32 ± 4.25 (20.00, 4.00)	(−)
Smoking period (years)	(−)	12.23 ± 8.65 (10.00, 9.50)	(−)
Average number of cigarettes smoked per day	(−)	15.46 ± 7.31 (15.00, 10.00)	(−)

*Note*: All data were reported as mean ± SD using Mann–Whitney sum tests and median with IQR. Significant differences that were describled in the text were highlighted in bold italic, with the symbol * representing significance level: *p < 0.05.

Abbreviations: Al, aluminum; BDI, Beck Depression Inventory; BMI, body mass index (calculated as weight in kilograms divided by height in meters squared); Cu, copper; Fe, iron; FTND, Fagerstrom Test for Nicotine Dependence; Li, lithium; Mg, magnesium; Mn, manganese; Pb, lead; PSQI, Pittsburgh Sleep Quality Index; SAS, Self‐Rating Anxiety Scale; Zn, zinc.

### 3.2 Correlation between CSF metal ion levels and scores in the general group

Spearman's correlations were performed to explore the relationship between the metal ions and scores in the general group (Table 2). Metal ions, including zinc, iron, lead, and aluminum, were found to positively correlate with depression measured by BDI scores (all *p* ≤ 0.01), and the correlation coefficients ranged from 0.209 to 0.281. As to sleep quality, metal ions, including zinc and lead, were found to have a positive correlation with PSQI scores (all *p* ≤ 0.05), and the correlation coefficients were below 0.200. There was no significant correlation between metal ions and SAS scores.

**TABLE 2:** Correlation of metal ions with scores in the general group

	SAS	BDI	PSQI	Mg	Zn	Fe	Pb	Li	Cu	Mn	Al
SAS	1										
BDI	0.233**	1									
PSQI	0.065	0.371**	1								
Mg	0.107	0.151	0.105	1							
***Zn***	−0.079	*0*.209**	*0*.184*	0.248**	1						
***Fe***	0.094	*0*.213**	0.145	0.294**	0.388**	1					
***Pb***	−0.042	*0*.267**	*0*.197*	0.255**	0.346**	0.496**	1				
Li	0.052	0.036	0.035	0.155*	0.149	0.188*	0.161*	1			
Cu	0.044	0.150	0.120	0.001	−0.050	−0.043	−0.010	0.078	1		
Mn	0.127	0.076	−0.047	−0.063	−0.020	−0.146	−0.059	−0.033	0.077	1	
***Al***	0.148	*0*.281**	0.143	0.140	0.103	0.121	0.209**	0.100	−0.045	0.084	1

*Note*: All data were reported as Spearman correlation analysis. Significant correlations that were describled in the text were highlighted in bold italic, with the symbol * and ** representing significance level: *p < 0.05, **p < 0.01.

Abbreviations: Al, aluminum; BDI, Beck Depression Inventory; Cu, copper; Fe, iron; Li, lithium; Mg, magnesium; Mn, manganese; Pb, lead; PSQI, Pittsburgh Sleep Quality Index; SAS, Self‐Rating Anxiety Scale; Zn, zinc.

### 3.3 Mediation analysis

Mediation models were performed according to the correlation (see above) and regression analyses (see below). To clarify whether the relationship between smoking and depression and the relationship between smoking and sleep quality is mediated by metal ions, four separate mediation models were conducted and only found the mediating effect of aluminum between smoking and depression (Table 3).

**TABLE 3:** Analysis of smoking and depression association with aluminum as a mediator

	Model1 (BDI)		Model2 (Al)		Model3 (BDI)	
	*β*	*t*	*β*	*t*	*β*	*t*
Age (years)	−0.0333	−0.431	−0.003	−0.375	−0.0276	−0.362
Education	−0.0263	−0.341	0.0286	0.363	−0.0319	−0.419
BMI	−0.0310	−0.403	−0.0364	−0.463	−0.0240	−0.317
Smoke	0.3242	4.129	0.2762	3.445	0.2708	3.381
Al	(−)	(−)	(−)	(−)	0.1935	2.510
*R* ^2^	0.322**		0.266*		0.373**	
*F* (*df*)	4.516 (4156)		2.977 (4156)		4.996 (5155)	

*Note*: All data were reported as mediation analysis.

Abbreviations: Al, aluminum; BDI, Beck Depression Inventory; BMI, body mass index (calculated as weight in kilograms divided by height in meters squared).

*p < 0.05, **p < 0.01.

First, we tested the effect of smoking on BDI scores after adjusting for age, years of education, and BMI, and the linear regression results showed that there was a positive effect of smoking on BDI scores (*R* ^2^ = 0.322, *β* = 2.403, *t* = 4.129, *p* < 0.01). Then we examined the effect of smoking on CSF aluminum after adjusting for age, years of education, and BMI, and the linear regression results showed that there was a positive effect of smoking on CSF metal ions (*R* ^2^ = 0.266, *β* = 0.576, *t* = 3.445, *p* < 0.05). Finally, we added mediator variables to test the mediating effect of smoking on BDI scores through aluminum, and linear regression results prove that the mediating effect of aluminum (*R* ^2^ = 0.373, *β* = 0.688, *t* = 2.510, *p* < 0.01).

**FIGURE 1:**
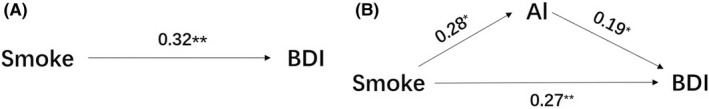
(A) Total effect between smoking and Beck Depression Inventory scores. (B) Effect decomposition of the mediation model for the relationship between smoking and BDI scores association with CSF aluminum (Al) as a mediator

The Bootstrap sampling method was used to explore the effect decomposition from mediation models. As shown in Table 4 and Figure 1, smoking had a direct effect on BDI scores (*z* = 0.05, 95% CI [0.001–0.113], *p* < 0.01), an indirect effect (*z* = 0.27, 95% CI [0.009–0.124], *p* < 0.05), and total effect (*z* = 0.32, 95% CI [0.001–0.169], *p* < 0.01). The results showed that the mediating effect of aluminum was incomplete mediation, and only about 1/6 (direct effect/total effect of the effect) of smoking on BDI scores was mediated by aluminum.

**TABLE 4:** Effect decomposition from mediation models

Effect decomposition	Estimated	95% CI		*p*
		Lower	Upper	
Direct effect	0.05	0.001	0.113	0.009
Indirect effect	0.27	0.009	0.124	0.013
Total effect	0.32	0.001	0.169	0.001

*Note*: All data were reported as mediation analysis.

Abbreviations: 95% CI, 95% confidence interval.

We also examined the mediating effects of zinc, iron, and lead on the relationship between smoking and BDI scores, there were no significant mediating effects (Zn, *z* = 0.01, 95% CI [−0.0875–0.1351], *p* = 0.64; Fe, *z* = 0.05, 95% CI [−0.0777–0.2074], *p* = 0.47; Pb, *z* = 0.03, 95% CI [−0.0497–0.1220], *p* = 0.47), which means their effects to depression were not caused by smoke. However, they might have another pathway effect on smoking and depression. Likewise, we examined the mediating effects of zinc and lead between smoking and PSQI, there was no significant mediating effect (Zn, *z* = 0.05, 95% CI [−0.0432–0.1394], *p* = 0.24; Pb, *z* = 0.05, 95% CI [−0.0563–0.1321], *p* = 0.46).

## 4 DISCUSSION

Our primary aim was to determine the associations of CSF metal ions with physiological and pathological aspects in smokers. Several epidemiological studies showed that smoking significantly negatively correlates with emotion regulation and sleep.^9^, ^30^ In this study, cigarette smoking was associated with depression and insomnia, as shown by higher BDI and PSQI scores, whereas there was no significant difference in SAS scores between the two groups. Moreover, significantly higher CSF levels of magnesium, zinc, iron, lead, lithium, and aluminum were found in active smokers. Additionally, some metal ions, including zinc, iron, lead, and aluminum, correlate significantly with the general group's BDI scores (all *p* ≤ 0.01). As for sleep, metal ions, including zinc and lead, were found to positively correlate with PSQI scores (all *p* ≤ 0.05) in the general group. Furthermore, there was no significant correlation between metal ions and SAS scores. Furthermore, mediation analysis showed that aluminum mediated the relationship between smoking and depression measured by BDI scores.

Previous research has indicated a strong association between smoking with depression and insomnia, and smoking might enhance the risk of mental illness.^31^, ^32^ Likewise, our results show higher BDI and PSQI scores in active smokers. Depression is an inflammatory disease.^33^ Short and long sleep duration and insomnia are standard among psychiatric populations and have previously been related to increased inflammation.^34^ Cigarette smoke contains thousands of chemicals,^35^ including free radicals, metals, tars, and others that induce inflammatory responses in body tissues, which may also increase one's predisposition to depression and insomnia.

Metal ions accumulate through tobacco smoke and cause neurotoxicity.^11^, ^36^, ^37^ Here, we found differences in CSF metal ions between nonsmokers and active smokers. Significantly higher CSF levels of magnesium, zinc, iron, lead, lithium, and aluminum were found in active smokers. A cytosolic accumulation of metal ions can lead to the mismetallation of proteins and cell death.^38^ Epidemiology and animal experiments demonstrated that many metal ions cause emotion regulation disorders and insomnia.^12^, ^13^ For example, studies showed that metal ions in serum increase the risk of anxiety, depression, and insomnia risk.^39^, ^40^, ^41^, ^42^, ^43^, ^44^ Furthermore, we found that smoking directly affected depression measured by BDI scores, and the relationship between smoking and depression was mediated by aluminum.

Aluminum is the most widely distributed metal in the environment and has interested many researchers over the past years. Previous human and animal studies supported the adverse effects of aluminum neurotoxicity, which are characterized by symptoms of depression.^45^, ^46^ A cross‐sectional study of Spanish schoolchildren, where investigators identified a negative association between aluminum levels and nondepressive symptoms.^47^ In this study, we found that CSF aluminum was associated with depression (Table 2). Several depression mechanisms are inseparable from oxidative stress and inflammatory responses. Depression is accompanied by inflammatory responses of the organism and consequent elevation of proinflammatory cytokines and increased lipid peroxidation.^48^ This finding is consistent with an animal study that aluminum might cause impairments in mitochondrial bioenergetics and the generation of oxidative stress, which might induce progressive multiregional neurodegeneration.^49^ Besides, the bioaccumulation of aluminum activates the IL‐1*β*/JNK signaling pathway, which leads to the death of rat hippocampal nerve cells and subsequent depression‐like behavior.^18^ Although the clinical and neuropathological effects of elevated brain aluminum levels have recently been described, there are few papers describing the consequences of excess emotion and sleep quality in active smokers.

In our study, a significantly higher CSF level of aluminum was found in active smokers than in nonsmokers, and the relationship between smoking and depression was mediated by CSF aluminum. Furthermore, the effect decomposition from mediation models showed that smoking‐induced depression is incompletely mediated by aluminum (Table 4 and Figure 1). That said, the direct effect of smoking on depression is established, but also through other effects, including cerebrospinal fluid aluminum levels. Smoking‐related cerebral inflammation might serve as a fundamental mechanism contributing to depression. Previous research has revealed that the accumulation of some inflammatory toxins from cigarette smoke activated classical microglia and further promoted depression.^50^ Therefore, whether other inflammatory toxins accumulate in cigarette smoke to mediate depression remains to be further investigated.

For mediation analysis, although our results showed that zinc, iron, and lead had no mediating effect, the spearman results showed that they were correlated with depression. This evidence suggests that these metal ions may influence depression through other pathways. Likewise, the spearman results showed that zinc and lead were correlated with insomnia measured by PSQI scores, which suggested that they might influence sleep quality through other pathways.

Along with these changes, active smokers showed significantly higher BMI compared to nonsmokers, indicating a weight‐gaining effect of cigarette smoking on the participants. Berhe et al.^51^ found that smoking might mediate the increase in BMI. Therefore, the link between metal ions and BMI index in smokers might be a worthy research direction.

### 4.1 Limitations

This study has some limitations. First, participants were patients with anterior cruciate ligament injuries instead of healthy individuals, which might appear to confound our results. Second, we only recruited man subjects because of the relatively few women smokers in China. The investigation of gender differences could be a key direction for future research.

## 5 CONCLUSION

Cigarette smoking was associated with depression and insomnia, as shown by higher BDI and PSQI scores. Significantly higher magnesium, zinc, iron, lead, lithium, and aluminum CSF levels were found in active smokers than in nonsmokers. Furthermore, CSF aluminum played a mediating role in the relationship between smoking and depression, which further confirmed its neurotoxicity.

## FUNDING INFORMATION

The following grants supported this work: The Technology Support Project of Xinjiang (2017E0267), The 10th Inner Mongolia Autonomous Region “Prairie excellence” Project, Natural Science Foundation of Xinjiang Province (2018D01C228), Outstanding Youth Science and Technology Talents of Xinjiang (2017Q007), Natural Science Foundation of China (81560229 & 81760252), Beijing Natural Science Foundation (7152074).

## CONFLICT OF INTEREST

We do not have financial interests or personal relationships that could have influenced the work reported in this paper.

## Data Availability

The data supporting this study's findings are available from the corresponding author upon reasonable request.
